# Role of Color Doppler Imaging in Early Diagnosis and Prediction of Progression in Glaucoma

**DOI:** 10.1155/2013/871689

**Published:** 2013-09-17

**Authors:** Fatima Jimenez-Aragon, Elena Garcia-Martin, Raquel Larrosa-Lopez, Jose M. Artigas-Martín, Pilar Seral-Moral, Luis E. Pablo

**Affiliations:** ^1^Radiology Department, Ciudad Real General University Hospital, 13005 Ciudad Real, Spain; ^2^Ophthalmology Department, Miguel Servet University Hospital, 50009 Zaragoza, Spain; ^3^Radiology Department, Miguel Servet University Hospital, 50009 Zaragoza, Spain

## Abstract

This longitudinal and prospective study analyzes the ability of orbital blood flow measured by color Doppler imaging (CDI) to predict glaucoma progression in patients with glaucoma risk factors. Patients with normal perimetry but having glaucoma risk factors and patients in the initial phase of glaucoma were prospectively included in the study and divided, after a five-year follow-up, into two groups: “Progression” and “No Progression” based on the changes in the Moorfields regression analysis (MRA) classification of Heidelberg retina tomograph (HRT). An orbital CDI was performed in all patients and the parameters obtained were correlated with changes in HRT. A logistic discrimination function (LDF) was calculated for ophthalmic artery (OA) and central retinal artery (CRA) parameters. Receiver operating characteristics curves (ROC) were used to assess the usefulness of LDFs to predict glaucomatous progression. A total of 71 eyes were included. End-diastolic velocity, time-averaged velocity, and resistive index in the OA and CRA were significantly different (*P* < 0.05) between the Progression and No Progression groups. The area under the ROC curves calculated for both LDFs was of 0.695 (OA) and 0.624 (CRA). More studies are needed to evaluate the ability of CDI to perform early diagnosis and to predict progression in glaucoma in eyes.

## 1. Introduction

Ultrasound is a classic diagnostic tool for the morphologic evaluation of ophthalmic pathology [1, 2]. The ability of Doppler ultrasound to obtain quantitative measurements of vascular flow opens up a new range of diagnostic possibilities, allowing statistical analysis, with a greater degree of diagnostic discrimination in pathologies such as glaucoma whose pathophysiology seems to involve vascular factors [3–5].

Primary open angle glaucoma, according to the World Health Organization (WHO), is currently the most frequent cause of preventable irreversible blindness in the world [6, 7]. It is caused by optic neuropathy characterized by acquired irreversible loss of retinal nerve fibers that comprise the optic nerve (ON). Glaucoma axonal loss occurs years before noticeable alterations of the visual field (VF) are detected [8, 9]. Once the alterations occur, VF loss is irreversible; therefore, early diagnosis of glaucoma is critical for preventing progressive vision loss.

Glaucomatous injury is insidious and difficult to structurally recognize until advanced disease is already present due to the wide range of normal variations in the optic papilla and the retinal nerve fiber layer (RNFL). The fact that the ON head perfusion is directly related to retrobulbar circulation [10, 11] that is directly accessible to ultrasound study makes color Doppler imaging (CDI) a potential tool for the evaluation of early changes in vascular flow related to glaucoma. The use of CDI has been validated already in the evaluation of moderate and advanced glaucoma patients, consistently detecting flow velocity alterations and increased resistive index in these patients in comparison with healthy controls [12–17]. Nevertheless, CDI value as a tool for early glaucoma diagnosis and progression has not been systematically studied. To our knowledge, only Calvo et al. [18] have included glaucoma suspects or early glaucoma patients. In both studies, the orbital CDI parameters have been correlated with glaucoma development or progression based on structural criteria (Heidelberg Retina Tomograph version 3, HRT3) periodically determined over a long period. We also have performed a logistic discrimination function (LDF) to predict progression using ophthalmic artery (OA) and central retinal artery (CRA) parameters. Zeitz et al. [19] and Martínez and Sánchez [20] studied CDI value predicting glaucoma progression including established glaucoma patients. In both studies the methods used to determine glaucoma progression were cup-to-disc ratio in conjunction with VF testing [19] and with VF changes [20].

The primary objective of our study was to analyze in a group of patients with glaucomatous risk factors possible differences in orbital blood flow (OBF) measured by CDI, between patients who remained stable and those who progressed. Secondarily we investigated which ocular vascular structures are most sensible to detect progression and possible modifications of flow patterns associated with major changes and their diagnostic value.

## 2. Methods and Materials

A 5-year prospective longitudinal study was designed to analyze the possible OBF changes detected by CDI in glaucoma risk patients and in an early stage of glaucoma. The protocol followed the tenets of the Declaration of Helsinki for biomedical research and was approved by the Institutional Review Board of our hospital, and a written consent was obtained from all participants.

### 2.1. Patients

The study included patients with normal standard automated perimetry (SAP) but having glaucoma risk factors based on the features of the papilla (as determined by clinical assessment of the optic nerve head (ONH); see definition below), or the presence of ocular hypertension (≥21 mmHg), and patients in the initial phase of the disease with discretely altered SAP (mean deviation (MD) less than −5 dB) [21]. Patients were prospectively recruited as part of ongoing studies within the “Early Glaucoma Diagnosis Program,” a prospective longitudinal study designed to evaluate optic nerve structure and visual function in glaucoma, conducted at our hospital.

Inclusion criteria were refractive error of less than 5 spherical diopters and 2 diopters cylinder, open anterior chamber angle, transparent ocular media, and best-corrected visual acuity of 20/40 or better. Exclusion criteria were history of ocular or neurologic disease, intraocular surgery within 3 months before enrollment in the study or through the follow-up period, diabetes, and current use of a medication that could affect visual field sensitivity such as corticosteroids, antirheumatic drugs (quinolines, indomethacin, and allopurinol), psychiatric drugs (phenothiazine, thioridazine, and chlorpromazine), and drugs used in cardiology (practolol, amiodarone, and digitalis glycosides).

### 2.2. Study Protocol

Participants underwent an ophthalmologic examination that included clinical history, biomicroscopy of the anterior segment by a slit lamp, visual acuity, gonioscopy, ultrasonic pachymetry, ophthalmoscopy of the posterior segment, Goldmann applanation tonometry, and at least two reliable standard automated VF tests. To define the glaucomatous risk factors based on the features of the papilla, the ONH was clinically assessed in all subjects by evaluating simultaneous stereophotographs of the optic disc. Clinical assessment of the ONH was performed after mydriasis (0.5% tropicamide; Alcon Laboratories Inc., Fort Worth, TX) by evaluating stereophotographs of the optic disc (Canon CF-60UV fundus camera; Canon Inc., Tokyo, Japan). The photographs were evaluated by two glaucoma specialists (A. F. and L. P.) blinded to the patients' identity and clinical history. Glaucomatous optic disc morphology was defined as diffuse neuroretinal rim narrowing with concentric enlargement of the optic cup, localized notching, or both. Any disagreement was resolved by consensus.

Topographic analysis of the ONH was performed using a confocal scanning laser ophthalmoscope, the HRT3 (Heidelberg Engineering, Heidelberg, Germany). The HRT3 software displays several windows in which the topographic results are detailed; one of them, the Moorfields regression analysis (MRA) classification [22, 23], compares the rim area with the predicted rim area for a given disc area and age, based on confidence limits of a regression analysis derived from an internal database. The optic disc is divided into 6 color-coded sectors (nasal superior, temporal superior, nasal inferior, temporal inferior, nasal, and temporal), and each sector is classified as “within normal limits” if the percentage of the rim falls within the 95% confidence interval (CI) (represented in green), “borderline” if the percentage of the rim is between 95% and 99.9% CI (represented in yellow), and “outside normal limits” if the result is greater than 99.9% CI (represented in red).

**Figure 1:**
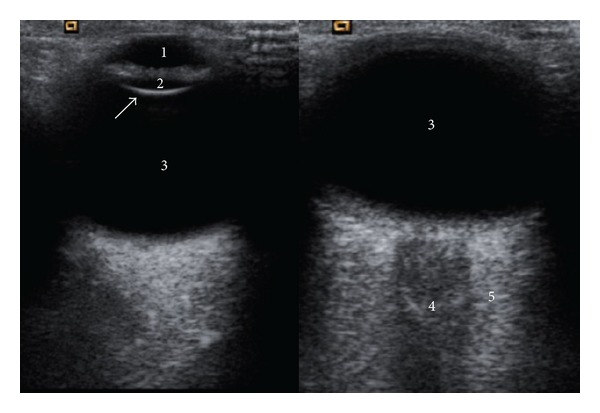
Transverse gray-scale US image that shows normal anatomy of ocular structures: anterior chamber (1), lens (2), vitreous body (3), in the conal region, the hypoechoic central band (4) corresponding to optic nerve sheath complex, and the retrobulbar fat (5).

OBF velocities of retrobulbar vessels were measured by color Doppler imaging at the beginning of the follow-up in all eyes. All the US examinations were performed with the same commercially available equipment (Siemens Sonoline Sienna, Erlangen, Germany) with a 7.5 MHz linear phased-array transducer. The same trained radiologist (F. J.) performed all tests. All subjects were examined in the supine position with the US probe gently placed on the closed upper eyelid taking care to minimize pressure on the globe, using a coupling gel. The ultrasound study began with a B-mode morphologic evaluation of the ocular structures in order to exclude the presence of concomitant injuries and to locate, in the conal region, the hypoechoic central band corresponding to ON, the reference place for the Doppler study of the ocular vessels (Figure 1).

**Figure 2:**
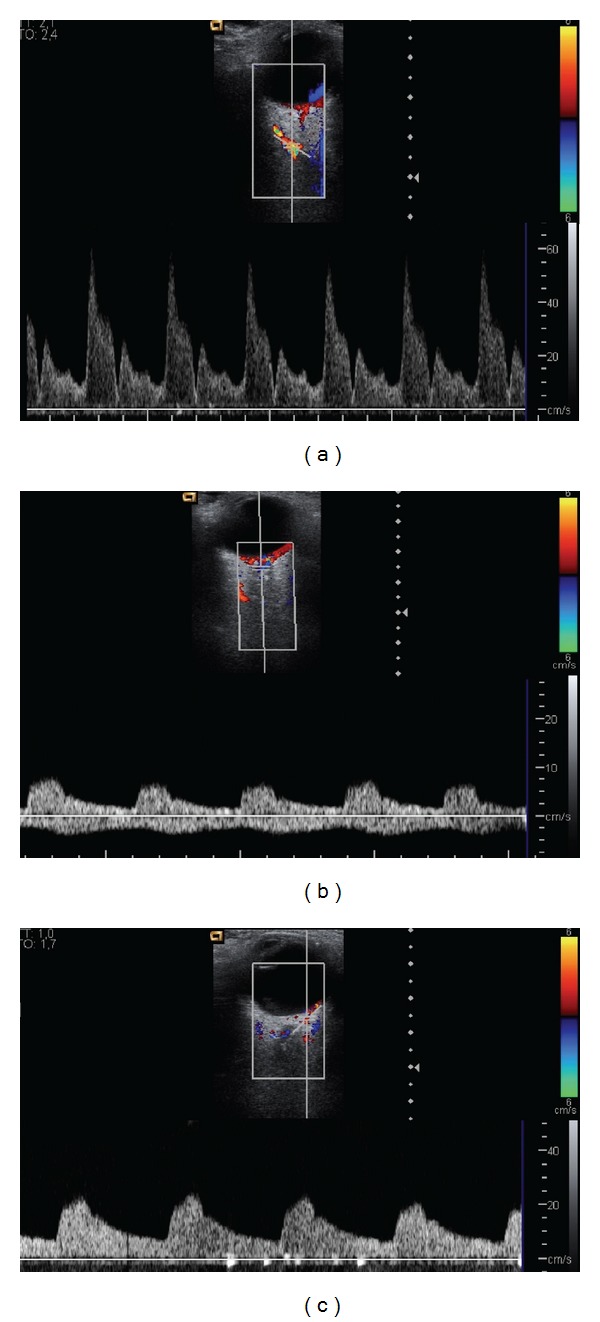
(a) Transverse color Doppler image that demonstrates the location of the ophthalmic artery (OA), medial to the optic nerve (ON), and the typical OA waveform obtained by pulsed Doppler that shows a sharp systolic peak, a dicrotic notch, and a relatively little flow in diastole. (b) Transverse color Doppler image showing the central retinal artery (CRA) and central retinal vein (CEV) in the center of the ON. The typical waveforms for central retinal vessels show the CRA curve above the zero axis (with rounded systolic peak and continuous flow during diastole) and the CRV below the zero axis (with low and continuous flow). (c) Transverse color Doppler image that demonstrates the posterior ciliary arteries (PCSAs) in the retrobulbar fat. The typical PCAs waveform obtained by pulsed Doppler shows a blunted systolic peak and a low to moderate flow velocity during diastole.

Later, CDI hemodynamic measurements of the OA, CRA, and the short posterior ciliary arteries (SPCAs) were obtained. The OA was studied in its nasal portion with regard to ON; CRA was identified next to central retinal vein as two parallel vascular structures with opposite flow sense included in the hypoechoic central band of the conal region; SPCAs were visualized in the posterolateral region of the papilla, on both sides, temporal and nasal, respectively (Figure 2).

Correction of the insonation angle was performed when the axis of the studied vessel was not enough aligned with the ultrasound beam, mainly in the ciliary arteries. The parameters evaluated included arterial peak systolic velocity (PSV), end-diastolic velocity (EDV), time-averaged velocity (TAV), resistive index [RI = (PSV − EDV)/PSV], pulsatility index (PI) [PI = (PSV − EDV)/TAV], and the relation systole/diastole (S/D) (S/D = PSV/EDV) [24, 25]. The operator was blinded to the results of all ophthalmology tests and the condition of each eye.

### 2.3. Classification into Groups and Follow-Up ****

*(a) Progression and No-Progression Groups Based on Topographic Criteria*. Patients were followed up for at least 5 years, with a minimum of once yearly reliable HRT3 examination. Conversion to glaucoma or progression was defined by a change of at least 3 sectors (from within normal limits to borderline, from borderline to outside normal limits, or from normal to outside normal limits) in the color-coded MRA classification at any moment during the 5 years of follow-up [26, 27]. Because the temporal and nasal sectors are generally less sensitive for detecting glaucomatous changes [23, 28], they were excluded from the statistical analysis.

As the sample included subjects with glaucoma risk factors but without damage as assessed by SAP as well as glaucoma patients with slightly altered SAP, the concept “glaucoma progression” included progression from no glaucoma damage to a certain grade of glaucoma and also from low-grade glaucoma to high-grade glaucoma. During follow-up time, each patient was treated at the discretion of the attending ophthalmologist.

*(b) Patient Classification Based on Perimetry and Glaucoma Risk Factors*. The sample was divided into three groups based on the VF, intraocular pressure, and the aspect of the papilla. These three groups were composed of

1. only patients with initial perimetric glaucoma,
2. patients with normal perimetry that were divided into two groups: patients with normal ONH but with intraocular hypertension and patients with normal intraocular pressure but with glaucomatous ONH. The baseline characteristics of these two groups were analyzed separately.

### 2.4. Statistical Analysis

All statistical analyses were performed using the IBM Statistical Package for the Social Sciences (SPSS 17.0, SPSS Inc., Chicago, IL, USA) and MedCalc (version 9.3 MedCalc Software, Belgium) statistical software. The Kolmogorov-Smirnov test was used to assess sample distribution. Because of the nonparametric distribution of data, differences between groups of patients were compared using the Mann-Whitney *U* test or ANOVA test for comparison of two or three variables, respectively. Chi-square test was used to compare qualitative categorical variables between both groups. Values of *P* < 0.05 were considered to be indicative of statistically significant differences.

The relative importance of each independent variable was assessed by stepwise binary logistic regression analysis using the forward Wald method. The Wald chi-square statistic tested the unique contribution of each independent variable (predictor variable), in the context of the other predictors (holding constant the other predictors), eliminating any overlap between them. In this study, the dependent variable was whether a patient glaucoma progressed or not, and the independent variables were PSV, EDV, TAV, RI, PI, and S/D for each artery. The significant parameters of the Doppler US study for each artery then were combined to generate a new variable, the logistic discrimination function (LDF), a score formed by taking a weighted sum of the predictor variables as LDF = *a* − *b* × *c*, where *a* is a constant of the model influences by all the studied parameters and *b* is the logistic regression coefficient of the more significant parameter (*c*).

The sensitivity and specificity of the LDF for each artery to predict glaucomatous progression were evaluated using receiver operating characteristics (ROC) curves. An area under the ROC curve (AUC) of 1.0 represents perfect discrimination, whereas an AUC of 0.5 represents chance discrimination.

## 3. Results

A total of 95 eyes of 95 consecutive subjects who fulfilled the inclusion criteria were prospectively preenrolled. Twenty-eight patients were excluded from the study due to noncompletion of required tests (17 patients), inability to perform at least one of the tests included in the study protocol (5 patients), and not providing informed consent (2 patients). Finally, 71 eyes from 71 patients of Caucasian origin were included in the statistical analysis.

As defined above, according to the changes in the MRA over the 5 years of follow-up, the sample was divided into 2 groups: Progression and No-Progression. The No-Progression group included 59 patients (83.1%), 24 males (40.67%) and 35 females (59.32%), and the Progression group included 12 patients (16.9%), 3 males (25%) and 9 females (75%). The clinical and demographic information of both groups at baseline, including sex, age, mean intraocular pressure (IOP), pachymetry (CCT), mean deviation of standard automated perimetry (MD of SAP), pattern standard deviation (PSD) of SAP and disc area, and vertical cup-to-disc ratio (C/D), is showed in Table 1. No significant differences were found between the two groups.

The clinical and demographic information was compared between the two groups of patients with normal perimetry, those with normal VF and normal ONH but with ocular hypertension, and those with normal VF and ocular pressure but with glaucomatous ONH. This information is shown in Table 2. No significant differences were detected between the two groups in age, sex, pachymetry, or perimetry. Significant differences were observed between groups in IOP, which is related to the classification criteria (first group included patients with intraocular hypertension and the second one included patients with normal IOP). The percentage of patients that progressed in each group was similar.

**Figure 3:**
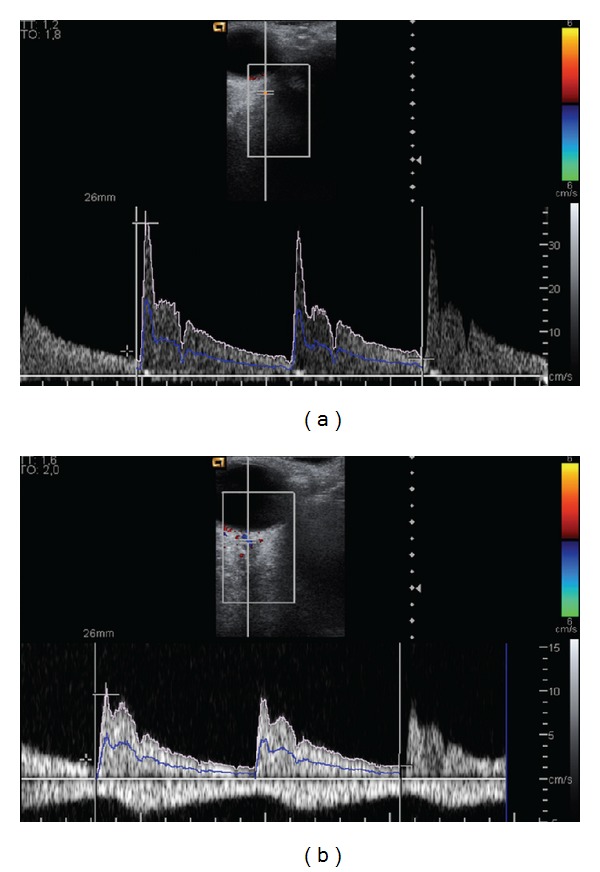
(a) Doppler frequency shift for the ophthalmic artery in the right eye of a patient that showed progression in the HRT-3 (peak systolic velocity PSV = 34.9 cm/s, end-diastolic velocity EDV = 3.7 cm/s, time-averaged velocity TAV = 10.1 cm/s, resistive index RI = 0.89, pulsatility index PI = 3.09, and the relation systole/diastole S/D = 9.43). (b) Doppler frequency shift for the central retinal artery in the right eye of the same patient (PSV: 9.6 cm/s; FDV = 1.4 cm/s; TAV = 3.6 cm/s; S/D = 6.86; RI: 0.85; PI = 2.28).

The hemodynamic measurements obtained at CDI in the Progression and No-Progression groups were compared, as shown in Table 3. Significant differences between groups were found in the OA and CRA, on EDV, TAV, and RI (*P* < 0.05) (Figure 3).

The OA and the CRA showed higher PSV and lesser PI in the No-Progression group than in the Progression one; the SPCAs showed higher flow velocities and lesser RI and PI in No-Progression group; however, these differences were not statistically significant.

As previously mentioned, statistically significant differences between the Progression and No-Progression groups were found in OA and CRA, so an LDF that allows maximized measurable differences between the groups was made for each one. The most significant parameters of the CDI study for each artery assessed by stepwise binary logistic regression were combined to generate an LDF for each artery obtaining OA LDF 1.106 − 0.228 × AO TAV and ACR LDF = −5.102 + 2.114 × ACR PI.

**Figure 4:**
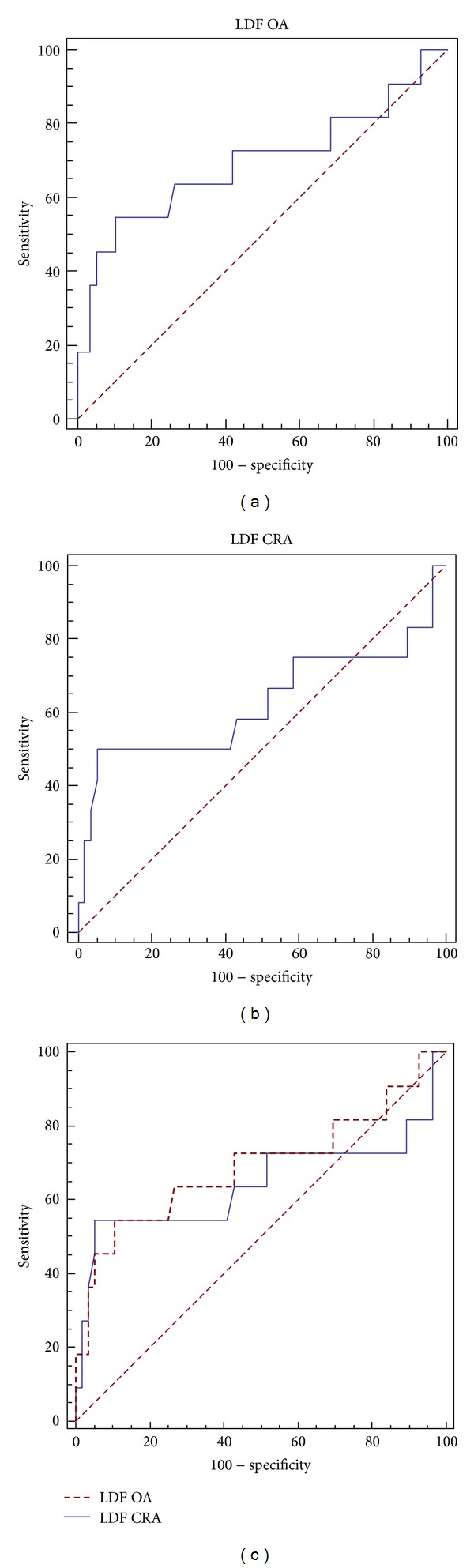
The ROC curve shows the diagnostic capability of the LFD for ophthalmic artery (a) and central retinal artery (b). The comparison between both ROC curves (c) shows best calculated cutoff points.

The diagnostic capability of both LDFs was calculated using ROC curves, obtaining an AUC of 0.695 for the OA (95% confidence interval (CI): 0.571–0.801) (Figure 4(a)) and 0.624 (95% CI: 0.5–0.737) for CRA (Figure 4(b)), being in both cases over 0.5. The comparison of both curves (Figure 4(c)) showed cutoff points with a specificity of 90% with a sensitivity of 54.55% in the OA and a specificity of 94% with a sensitivity of 50% in the RCA.

As previously described, the sample was also divided into groups based on perimetry and glaucoma risk factors, independent of progression. The orbital CDI characteristics of these groups were also compared and the results are shown in Table 4. These groups comprised (1) patients with initial perimetric glaucoma, (2) patients with initial perimetric glaucoma and those with normal VF and ONH but having ocular hypertension, (3) the whole population. Flow velocities were larger and resistance indices (RI, PI, and S/D) smaller in the second group than in the other two groups. There were no significant differences between groups, however, in any hemodynamic parameter.

## 4. Discussion

The alteration in OBF dynamics is well recognized in glaucoma. Many studies over the last twenty years have shown that vascular factors may play an important role in glaucoma pathogenesis due to an OBF autoregulation failure [3, 4]. Moreover, although elevated IOP is a well-known major risk factor for glaucoma, it has been demonstrated that there are numerous patients in whom glaucoma progressed despite an IOP therapeutic reduction [29, 30], so IOP is a poor progression marker. In the present study no significant differences in IOP (registered at the beginning of the study) were observed between glaucoma Progression and No-Progression patients; however, significant differences were found for some orbital CDI parameters between both groups. A combination of decrease in flow velocities and increase in pulsatility and resistive indices obtained by orbital CDI, was registered in progressing glaucoma patients compared to those who remain stable. These results suggest that orbital hemodynamics studied by CDI may represent an important biomarker to discriminate glaucoma patients with higher risk for progression. Doppler US may thus help to institute a more aggressive clinical management in conflicting cases with higher progression risk. Although the current data does not allow us to establish hard velocity parameters to define the threshold between normal and pathologic dynamics, it does show a recognizable difference in the mean velocities of the two groups, but with an overlap between the two patient groups due to the wide range of measured values, which is also typical of all other functional and structural glaucoma tests currently available in clinical practice. However, Calvo et al. [18] determined after a follow-up of 48 months that an RI value higher than 0.75 in the OA was associated with glaucoma progression in glaucoma suspects.

Doppler US has been recognized in many papers as an effective tool to assess alteration in these flow dynamics [12, 13, 15]. Most of published studies include healthy patients without glaucoma risk factors as controls and patients with well-established glaucomatous damage as cases, while our population was performed by subjects with risk factors for glaucoma, but without yet established visual field impairment (<−5 dB of MD). Although this population selection may exhibit less apparent differences in retrobulbar flow dynamics, it may validate better the usefulness of Doppler US in early glaucoma diagnosis, which is very relevant in glaucoma to prevent the irreversible damage of RNFL.

The orientation of the study towards early diagnosis of glaucoma also influenced the diagnostic method used to establish the subjects' progression, opting for a structural method, specifically the MRA OF HRT3, instead of perimetry changes. Previous studies [31–33] demonstrated that optic nerve or retinal nerve fiber layer damage precedes visual field damage in glaucoma, so we used topographic analysis of the optic nerve and retinal nerve fiber layer to determine progression in our sample.

Two of the six sectors in which the HRT3 divides the papilla, the temporal and nasal sectors, have less sensitivity for detecting glaucomatous changes [23, 28]. The superior and inferior sectors of the ONH, however, have been used to assess early signs of glaucoma progression [34, 35]. This could be related to the thicker retinal nerve fiber layer bundles in the superior and inferior regions and the thinner bundles in the temporal and nasal regions, which allows the HRT to more easily detect and quantify changes in the vertical axis. Based on these data and to improve accuracy and avoid bias, the temporal and nasal sectors were excluded from the statistical analysis.

As Plange et al. reported [15], we found higher flow velocities and lesser RI and PI in SPCAs in the group of No-Progression, but these differences were not statistically significant. In contrast, there are some studies that show reduced circulation in these vessels in patients with glaucoma [36–39], and Zeitz et al. [19] found decreased blood flow velocities in the SPCA associated with glaucoma progression. The small caliber of these vessels avoid individual measurements, and due to their direction, variable insonation angles are usually required for their analysis. Furthermore, the wide variability in their measurements is higher than in other vessels and has been suggested previously [40–42]. On the other side, the larger size and more accessible locations of the OA and the CRA make their measurements easier and more reproducible with Doppler US. Vascular supply of the external part of the retina depends directly on the SPCA, branches of the OA, making it therefore presumable that factors affecting the flow parameters of OA and CRA may also similarly impact the SPCA (whose direct demonstration is technically more difficult). For these reasons, we think that larger studies may find significant alterations in the CDI parameters of SPCAs. Other authors [16, 18] did not find differences between patients with stable and deteriorating visual fields for the CRA. The different designs and samples of the studies, as well as the different techniques used, make comparison of results difficult. We found significant differences in some CDI parameters, as previous authors have reported. RI has some advantages over other parameters because it includes systolic and diastolic velocity values and is the most reproducible parameter in Doppler ultrasound (coefficient of variation around 6%) [36]. CDI is a noninvasive method that allows the analysis of the vascular implications in glaucoma. The reproducibility and accuracy of OBF measurements are variable and depend on technique homogeneity and methodologic design [41].

The purpose of the LDF for the OA and CRA was to reflect the best combination of CDI parameters that may differentiate between stable and progressive glaucomatous patients and predict glaucoma progression.

In the present study, both LDFs showed an AUC higher than 50% (AUC of 0.695 for the OA and 0.624 for CRA). Specificities of our LDFs ranged between 90% and 94%, and sensitivity was 55%. This is a relatively poor diagnostic ability, in part, due to the type of sample used to focus on early diagnosis, where the onset of visual field loss was not yet severe. A sample with more severe disease may have provided increased sensitivity, as Plange et al. found in glaucoma patients [12]. These results suggest that the use of the proposed LDFs and orbital Doppler ultrasound should be associated with other diagnostic methods to establish glaucoma progression. The orbital hemodynamics studied by CDI, however, seem to be a useful biomarker for predicting the probability of glaucoma progression, especially for cases with nonclassical behavior or in the early stages for whom the results could lead to the adoption of more or less aggressive therapeutic measures.

We must acknowledge some limitations of our study. Orbital CDI was only assessed at the beginning of the follow-up; however, other orbital CDI registers in the middle and at the end of the study could correlate strongly the association between orbital CDI and glaucoma progression. The relationship between CDI and clinical characteristics was also only analyzed at the beginning of the follow-up, and, although no significant differences were found between them in both groups of patients, other factors not included in the initial evaluation or changes in the parameters included could have taken part in glaucoma progression. In addition, glaucoma is a chronic disease with a long period of evolution, for which the follow-up that has been used in this study is a period that allows us to extract initial conclusions, to detect design mistakes and to raise new hypotheses, though it is not still long enough to assume etiopathogenic correlations and long-term predictions.

Although the initial patient selection (patients with normal VF and glaucoma risk factors or patients with slightly altered VF) allowed us to orient the results to early glaucoma diagnosis focusing on glaucoma progression, these aspects could have some limitations, such as the potential inclusion of patients with normal tension glaucoma (NTG) in the same category as normal subjects with asymmetric discs. For this reason, we performed another analysis by dividing patients according to the perimetry, IOP, and ONH aspects. In this analysis, the CDI characteristics differed between groups, with larger flow velocities and smaller resistance indices in the group that included patients with normal perimetry and normal intraocular pressure but with glaucomatous ONH compared with those with only perimetric glaucoma or the whole study population. This could be due, at least in part, to the potential association of perimetric damage with smaller Doppler velocities in all orbital vessels studied, although the differences were not significant. Additional studies with larger groups of patients are needed to evaluate this tendency.

Another potential limitation of the study is the absence of a control group of totally normal subjects.

In conclusion, our work suggests that orbital hemodynamics studied by CDI may be useful as a biomarker to predict glaucoma progression, especially in OA and CRA whose LDFs reached high specificities. Further studies with a larger population are needed. An effort should be made by researchers to determine uniform methodologic standards of orbital Doppler US, in order to be able to compare results.

*Practical Applications*. As retrobulbar hemodynamic alteration might represent a risk factor for glaucoma progression even in an early stage, when visual field is still normal and when a classical risk factor as IOP is not altered, orbital CDI would constitute an important diagnosis method, whose results could help to adopt more or less aggressive therapeutic measures in conflicted cases.

Further Doppler studies are needed to determine the extent to which the parameters can detect glaucoma in early stages of the disease or to monitor treatment effectiveness. Doppler measurements in glaucoma can be useful in combination with other parameters and clinical explorations.

## Figures and Tables

**Table 1:** Clinical and demographic information of both study groups, Progression and No-Progression; *P* < 0.05 was considered statistically significant.

	Unit	No progression	Progression	*P *
Number of eyes		59	12	n/a
Sex	Male/female	24/35	3/9	0.546
Age	Years (SD; range)	54.03 (8.911; 24–76)	55.75 (15.16; 27–71)	0.876
Mean IOP	Hg mm (SD; range)	23.58 (1.50; 21–26)	22.22 (1.47; 21–25)	0.124
Pachymetry (CCT)	*µ*m (SD; range)	550.80 (38; 390–636)	532.42 (34.47; 465–596)	0.347
MD of SAP	Decibels (SD; range)	−1.79 (3.06; −17.37–1.35)	−0.78 (0.77; −2.07–0.51)	0.656
PSD of SAP	Decibels (SD; range)	2.22 (2; 0.92–12.81)	1.8 (0.69; 1.17–3.24)	0.076
C/D	Mean (SD; range)	2.75 (0.92; 1–5)	3.92 (0.67; 3–5)	0.844
Spherical equivalent	Diopters (SD; range)	−0.56 (2.19; −3.97–2.81)	−0.78 (2.37; −3.33–2.55)	0.396

IOP: intraocular pressure; CCT: central corneal thickness; MD of SAP: mean deviation of standard automated perimetry; PSD of SAP: pattern standard deviation of standard automated perimetry; C/D: vertical cup-to-disc ratio in stereophotographs.

**Table 2:** Clinical and demographic information of both groups of normal perimetry patients. *P* < 0.05 was considered statistically significant.

	Patients with normal perimetry, normal optic nerve head, and intraocular hypertension (*n* = 45)	Patients with normal perimetry, glaucomatous optic nerve head, and normal intraocular pressure (*n* = 16)	*P *
Sex (male/female)	17/28	6/10	0.679
Mean age (years)	55.41 ± 13.5	53.98 ± 16.00	0.107
Mean IOP (Hg mm)	26.01 ± 10.43	14.98 ± 7.78	*0.009 *
Mean Pachymetry (*μ*m)	543.21 ± 35.56	545.09 ± 32.33	0.254
MD of SAP (decibels)	−1.55 ± 2.34	−1.12 ± 2.98	0.330
Progression in MRA during 5 years (*n*)	9	3	0.412

IOP: intraocular pressure; MD of SAP: mean deviation of standard automated perimetry.

**Table 3:** Comparison of hemodynamic parameters obtained at color Doppler imaging in Progression and No-Progression groups.

Doppler US parameter	Unit	Progression group	No-Progression group	*P *
OA Ophthalmic artery				
PSV	cm/s (SD)	25.68 (9.9)	30.70 (8.14)	0.163
EDV	cm/s (SD)	5.29 (2.83)	7.32 (3.01)	**0.043**
TAV	cm/s (SD)	10.64 (5.34)	14.4 (4.70)	**0.042**
RI	(SD)	0.79 (0.05)	0.75 (0.05)	**0.038**
PI	(SD)	1.92 (0.58)	1.66 (0.33)	0.093
S/D	(SD)	5.17 (1.32)	4.50 (1.15)	0.091
CRA Central retinal artery				
PSV	cm/s (SD)	8.79 (2.92)	9.56 (2.31)	0.099
EDV	cm/s (SD)	1.88 (0.54)	2.41 (0.84)	**0.034**
TAV	cm/s (SD)	3.92 (1.28)	4.6 (1.25)	**0.042**
RI	(SD)	0.77 (0.06)	0.73 (0.05)	**0.043**
PI	(SD)	1.85 (0.59)	1.51 (0.29)	0.180
S/D	(SD)	4.66 (1.51)	4.12 (1.03)	0.261
SPCAs Short posterior ciliary arteries				
PSV	cm/s (SD)	15.12 (4.07)	16.32 (6.52)	0.914
EDV	cm/s (SD)	4.32 (1.81)	4.90 (2.07)	0.349
TAV	cm/s (SD)	8.24 (2.88)	8.47 (3.30)	0.994
RI	(SD)	0.72 (0.08)	0.69 (0.06)	0.341
PI	(SD)	1.40 (0.39)	1.12 (0.25)	0.457
S/D	(SD)	3.90 (1.38)	3.43 (0.74)	0.618

Values are expressed as mean (standard deviation). Arterial evaluations included peak systolic velocity (PSV), end-diastolic velocity (EDV), time-averaged velocity (TAV), resistive index (RI), pulsatility index (PI), and the relation systole/diastole (S/D). *Significant differences (*P* < 0.05) between both groups were found in the OA and CRA, on FDV, TAV, and RI (in bold print).

**Table 4:** Comparison of hemodynamic parameters obtained by color Doppler imaging in the following three groups: A—patients with perimetric glaucoma, B—patients with perimetric glaucoma and patients with normal visual field and intraocular pressure but with glaucomatous optic nerve head, and C—the whole study population.

Doppler US parameter	Unit	Patients with perimetric glaucoma (*n* = 10)	Patients with perimetric glaucoma + patients with normal VF and IOP but with glaucomatous ONH (*n* = 55)	The whole population (*n* = 71)	*P *
OA Ophthalmic artery					
PSV	cm/s (SD)	25.60 (10.21)	29.87 (8.79)	27.43 (9.12)	0.234
EDV	cm/s (SD)	5.44 (2.92)	7.28 (2.89)	6.21 (2.77)	0.134
TAV	cm/s (SD)	10.52 (5.10)	14.33 (4.81)	12.01 (5.01)	0.459
RI	(SD)	0.78 (0.05)	0.76 (0.04)	0.77 (0.05)	0.501
PI	(SD)	1.93 (0.48)	1.65 (0.37)	1.79 (0.40)	0.192
S/D	(SD)	4.98 (1.28)	4.72 (1.21)	4.73 (1.22)	0.237
CRA Central retinal artery					
PSV	cm/s (SD)	9.00 (2.67)	9.11 (2.44)	9.07 (2.52)	0.109
EDV	cm/s (SD)	1.91 (0.63)	2.14 (0.62)	2.01 (0.62)	0.207
TAV	cm/s (SD)	4.03 (1.26)	4.39 (1.27)	4.16 (1.27)	0.119
RI	(SD)	0.76 (0.05)	0.74 (0.05)	0.75 (0.05)	0.096
PI	(SD)	1.79 (0.50)	1.58 (0.32)	1.67 (0.37)	0.102
S/D	(SD)	4.66 (1.40)	4.21 (1.29)	4.49 (1.33)	0.440
SPCAs Short posterior ciliary arteries					
PSV	cm/s (SD)	15.54 (4.58)	15.96 (5.49)	15.87 (5.03)	0.450
EDV	cm/s (SD)	4.50 (1.70)	4.70 (1.97)	4.62 (1.88)	0.691
TAV	cm/s (SD)	8.37 (2.87)	8.45 (3.13)	8.43 (2.99)	0.409
RI	(SD)	0.72 (0.07)	0.70 (0.08)	0.71 (0.07)	0.631
PI	(SD)	1.37 (0.32)	1.20 (0.30)	1.26 (0.31)	0.422
S/D	(SD)	3.88 (1.15)	3.61 (0.98)	3.69 (1.00)	0.702

Visual field (VF); intraocular pressure (IOP); optic nerve head (ONH); values are expressed as mean (standard deviation). Arterial evaluations included peak systolic velocity (PSV), end-diastolic velocity (EDV), time-averaged velocity (TAV), resistive index (RI), pulsatility index (PI), and the relation systole/diastole (S/D). *Significant differences (*P* < 0.05).
